# Primary Prevention of Colorectal Neoplasias: Ample Evidence, Lack of Guidelines

**DOI:** 10.14740/jocmr1772w

**Published:** 2014-03-31

**Authors:** Fotios Seretis, Charalampos Seretis, Nikolaos Liakos

**Affiliations:** a Department of Internal Medicine, Medical School of University of Patras, Greece; b The 2nd Department of General Surgery, 401 General Army Hospital of Athens, Greece

## Introduction

Colorectal cancer (CRC) is the third most commonly diagnosed type of malignancy in males and the second in females, with over 1.2 million new cancer cases and 608,700 deaths estimated to have occurred in 2008 [1]. Sporadic CRC accounts for 75% of the total CRC cases, with colorectal adenoma regarded as the precursor lesion (adenoma-carcinoma sequence) [2]. Despite the major advances in the screening methods, treatment options and follow-up frameworks, there is ample evidence highlighting the preventability of colorectal neoplasias by the implementation of appropriate lifestyle modifications, with the most extensively studied parameters being the effects of physical activity, specific dietary patterns, smoking and alcohol consumption [3]. Moreover, the chemoprevention of colorectal neoplasias is an area of ongoing research, focusing on the potentially preventive role of certain drugs and micronutrients [4].

Although there is now solid evidence regarding the clear protective role of lifestyle modification in terms of preventing colorectal neoplasias, there is lack of specific guidelines from the leading health organizations and professional bodies, with the currently available guidelines providing general rather specific frameworks for establishing national preventive policies. Herein, we attempt to pinpoint in a best evidence topic frame the key findings in the field of primary prevention of colorectal neoplasias, underlining the necessity of a qualitive turn towards setting specific lifestyle modification targets instead of general guidance.

## Physical Activity

Increased physical activity is considered to be an important preventive factor concerning the risk of colorectal neoplasias, mainly through modifying the metabolic profile (hyperinsulinemia, over-expression of insulin growth factors), reducing the body mass index, decreasing the gut transit time and enhancing the anti-cancerous properties of the immune system. However, the exact mechanisms that regulate the above mentioned interactions have not yet been completely elucidated.

A recent meta-analysis revealed the presence of an inverse association between physical activity and colon polyps in both sexes (risk reduction by 15%) [5]. Similarly, there is solid evidence regarding the beneficial role of physical activity in the prevention of CRC [6, 7], which appears to apply for both proximal and distal colon lesions [3]. Although the heterogeneity of the current studies poses certain limitations in determining a threshold of physical exercise that would reduce significantly the risk of colorectal neoplasias, it seems that its protective effects are exhibited even at the extent of regular leisure-time physical activity, with a reduction of colon cancer risk up to 20% [8].

## Dietary Patterns

The adoption of a western-type dietary pattern has for long been considered as one of the most significant risk factors for the development of colorectal neoplasias; hence, the reduction of CRC risk after the implementation of qualitive and quantitive modifications of the dietary intake has been extensively investigated.

According to a recent meta-analysis evaluating the existence of a nonlinear association of fruit and vegetable intake with CRC risk, the daily consumption of 100 - 200 g of vegetables was found to reduce CRC risk about 10%, while consumption of 600 g of fruits daily resulted in a similar risk reduction of 15% [9]. With respect to the intake of dairy products, the reduced risk is suggested to be most pronounced at the higher levels of intake, equivalent to 2 - 3 glasses of milk per day, with the latter applying for colon but not for rectal cancer [10]. Interestingly, in contrast with the current perception, no firm association has been established between the overall dietary fat intake (animal and plant fat) and CRC risk, as described in a meta-analysis of 13 prospective cohort studies [11]. Regarding the preventive role of dietary fiber, recent data indicate the existence of 10% reduction of the risk of CRC for each 10 g/day intake of total dietary fiber and cereal fiber and about a 20% reduction for each three servings (90 g/day) of whole grain daily and further reductions with higher intake [12]. On the contrary, the risk increase for the development of CRC, as estimated in linear dose-response models, is 14% for every 100 g/day increase of total red and processed meats, 25% in colon cancer and 31% in rectal cancer [13]. Moreover, while Xu et al, in their recently published meta-analysis, demonstrated the lack of any association between the risk of development colorectal adenomas and the dietary intake of white meat, poultry and fish [14], Wu et al, in a meta-analysis of 22 prospective cohort and 19 case-control studies, concluded that fish consumption may reduce the risk of CRC by as much as 12% [15]. The latter was also supported by the findings of Geelen et al, who indicated a decrease of the risk of CRC for each extra time that fish was consumed per week and for each extra 100 g of fish intake per week (4% and 3%, respectively), failing, however, to associate it with differences in the intake of n-3 fatty acids [16]. Finally, the available data cannot find the existence of an independent association between diets high in carbohydrate, glycemic index, or glycemic load and CRC risk [17].

## Smoking and Alcohol

Current smokers have a modestly higher risk of developing CRC, as demonstrated in the meta-analysis of Tsoi et al [18] than never smokers, with the risk being more significant for males rather than females. In the same study, rectal cancer was more closely related to smoking than colonic cancer. Finally, it appeared that ex-smokers still carried a higher CRC risk than never smokers. The increased risk of CRC was related to cigarettes per day, longer years of smoking, or larger pack-years.

Similarly, a causal relation has been proven to exist between high intake of alcohol and increased risk for CRC, providing additional evidence of an association for moderate intake of alcohol and a shape for the dose-risk relationship [19]. Compared with nondrinkers or occasional alcohol drinkers, moderate drinking (> 0.6 - 49.9 g/day of ethanol) was associated with a 21% and heavy drinking (≥ 50 g/day of ethanol) with a 52% increased risk for CRC, whereas light alcohol consumption (≤ 12.5 g/day of ethanol) was not associated with an increased risk. However, results of the dose-risk analysis showed a statistically significant 7% increased CRC risk for 10 g/day of alcohol intake, which includes light alcohol consumers.

## Chemoprevention

Currently, chemoprevention is one of the most debated issues in the framework of primary prevention of colorectal neoplasias, with the most well-investigated agents being aspirin and other NSAIDs in terms of commonly used drugs and calcium, vitamin D, folic acid and antioxidants (including vitamin A, vitamin C, vitamin E, selenium and beta-carotene) in terms of micronutrients. Concerning the first group, Cooper et al, in their study evaluating the clinical effectiveness and cost-effectiveness of drug and micronutrient interventions for the prevention of CRC and/or adenomatous polyps [4], found that aspirin and celecoxib may reduce the recurrence of adenomas and incidence of advanced adenomas in individuals with an increased risk of CRC; moreover, the researchers concluded that COX-2 inhibitors may decrease polyp number in patients with FAP and also pointed that long-term use of aspirin probably reduces the incidence of CRC in the general population. However, the serious adverse effects of both pose significant limitations that currently prohibit their *a priori* induction in colorectal neoplasias chemoprevention schemes in the daily practice. Finally, in the same study, the researchers suggest that calcium supplementation could reduce the recurrence of adenomas in patients with increased risk of developing CRC, but no similar conclusion was drawn with respect to the general population.
